# Anesthetic Management of a Patient with DRESS Syndrome for Renal Transplantation

**Published:** 2016-11-01

**Authors:** P. G. Kadam, R. Nama, M. P. Modi

**Affiliations:** Department of Anesthesiology and Critical Care, Institute of Kidney Diseases and Research Centre and Institute of Transplantation Sciences, Ahmedabad, Gujarat, India

**Keywords:** Drug hypersensitivity syndrome, Renal transplantation, Eosinophilia, Hypersensitivity, Drug eruptions, Fever

## Abstract

Drug reaction with eosinophilia and systemic symptoms (DRESS) syndrome is a rare but potentially life-threatening hypersensitivity reaction characterized by widespread erythematous skin eruptions with fever, lymphadenopathy and visceral involvement—hepatitis, nephritis, pericarditis, and pneumonitis. There are numerous reports describing the management of such patients in intensive care units but hardly any describing the intraoperative anesthetic management of such patients. Herein, we report on a patient with DRESS syndrome who was scheduled for renal transplantation. The main goal in this case was to prevent a hypersensitive drug reaction intraoperatively and develop a safe alternative anesthesia plan for the patient. After pre-operative skin and intradermal tests, we chose the drugs that could be safely used for anesthesia. Usually general anesthesia is preferred for renal transplantation but in this patient we opted for combined spinal epidural anesthesia. Precautions that are to taken in such a case and the anesthetic management are discussed in detail herewith.

## INTRODUCTION

Drug reaction with eosinophilia and systemic symptoms (DRESS) syndrome is a rare but life-threatening condition characterized by skin rash, fever, lymphadenopathy, and internal organ involvement—hepatitis, nephritis, and pneumonitis. There are innumerable reports describing the management of these patients in critical care units but to our knowledge, this is the first report on the intraoperative anesthetic management of a patient with DRESS syndrome scheduled for renal transplantation.

## CASE REPORT

**Figure 1:**
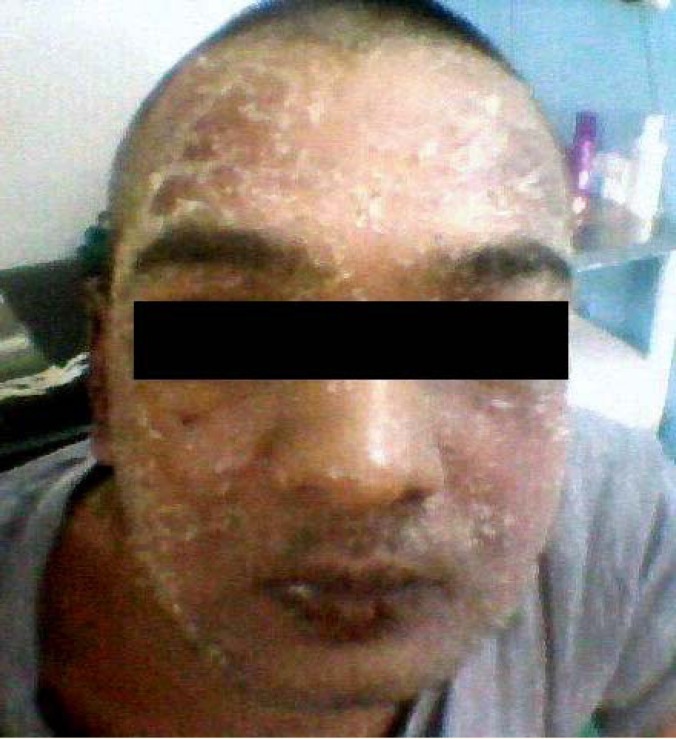
Intense scaling and edema over the patient's face

A 26-year-old man with end-stage renal disease was scheduled for renal transplantation. During his preanesthetic checkup it was noted that he had DRESS syndrome in response to antibiotics that had been prescribed for a catheter-related infection. He had high grade fever and erythematous maculopapular rash all over the body with intense scaling and edema over the face (Fig 1). All the investigations were normal except for leukocytosis (WBC 23,000/mm^3^) with 10% eosinophils and an abdominal CT showing reactive retroperitoneal lymphadenopathy and hepatomegaly. Dermatological opinion confirmed the diagnosis of DRESS after noting elevated serum LDH and IgE levels [1]. All the antibiotics were discontinued. He became afebrile with resolution of skin rashes. He was scheduled for renal transplantation under regional anesthesia. Prior to the surgery, skin prick and intradermal tests were done with antibiotics, lignocaine, bupivacaine, ropivacaine, fentanyl, and tramadol—the drugs most likely to be used during regional anesthesia according to standard protocols [2]. After confirming negative results, the drugs were used. He was dialyzed before his surgery. All his preoperative investigations were normal. In the operation theatre, the right internal jugular vein was cannulated for central venous pressure measurement. Epidural catheter was placed in the L_3-4_ interspace and kept 6 cm inside the epidural space. A test dose of lignocaine+adrenaline was given epidurally. Spinal anesthesia was given in L_4-5_ interspace with Quincke’s needle; 17.5 mg bupivacaine heavy was given after free flow of CSF to achieve a T_6_ sensory level. Intra-operative monitoring included temperature, pulse, NIBP, ECG, CVP, and urine output. We also remained alert for signs and symptoms of anaphylaxis including skin rash, tachycardia, hypotension, bronchospasm, and edema of lips or tongue. Epidural mixture of 7 mL of bupivacaine 0.5% and 7 mL of lignocaine 2% was given epidurally after 90 min of spinal anesthesia. Supplemental doses of local anesthetics were given epidurally hourly until the end of the surgery.

Three liters of normal saline was given and CVP was kept between 12 and 15 mm Hg. Intra-operatively, 500 mg methylprednisolone and 100 mL mannitol 20% were given just before release of the vascular clamps. Anastomosis time was 20 min and urine output was established immediately on release of the vascular clamps. The patient remained stable throughout the surgery. Post-operatively, the patient was transferred to ICU for further observation. Tramadol plus 0.125% bupivacaine were given epidurally for post-operative pain relief.

## DISCUSSION

DRESS syndrome is a hypersensitivity reaction characterized by erythematous skin eruptions with fever, lymphadenopathy and visceral involvement. It is associated with some blood alterations like eosinophilia and mononucleosis [1, 3]. Anticonvulsants, sulfonamides, phenobarbital, certain antibiotics, and NSAIDS are the common culprit drugs. The pathology of DRESS syndrome remains unclear but a defect in detoxification of causative drugs, immunological imbalance, and infection such as human herpes virus type 6 has been suggested. The most important steps in managing these patients are recognizing the presence of this syndrome and immediately stopping the offending drug.

Anesthesia represents a pharmacologically unique situation during which patients are exposed to multiple foreign substances like analgesics, antibiotics, antiseptics, blood products, heparin, neuromuscular blocking agents, and intravenous volume expanders, which can produce hypersensitivity reactions. The challenge in this case was to prevent a hypersensitivity drug reaction during renal transplantation because accompanying infections and internal organ involvement increase the morbidity and mortality in patients with DRESS syndrome. There are case reports where DRESS syndrome has caused interstitial nephritis, ARDS, thrombocytopenia, sepsis-like picture, and facial and tongue edema that need mechanical ventilation [3-5]. Any of these events in the post-transplantation period can make the prognosis grave for the patient as well as the renal allograft. Therefore, we made a list of all the antibiotics that the patient was exposed to and those were not used during the peri-operative period. We routinely give albumin to increase the CVP during transplantation, however, it was avoided in this case because 4% of hypersensitivity reactions under anesthesia occur with the use of colloids [6]. We maintained the CVP with crystalloids only. We remained alert for signs of anaphylaxis and basic and advanced cardiac life supports were kept ready. 

The next task was to decide which type of anesthesia would be favorable in this case. We did not opt for general anesthesia because here patients are exposed to multiple drugs which increase the probability of hypersensitivity reactions. Muscle relaxants are involved in 70% of the hypersensitivity reactions under anesthesia [6, 7]. As our patient had end-stage renal disease, the choice of muscle relaxants were atracurium and cis-atracurium, both of which are known to cause hypersensitivity reactions and thus were not suitable. Recognition of anaphylaxis under general anesthesia may be delayed. Moreover, there have been reports where patients with eosinophilia, when exposed to general anesthesia, developed complications like ARDS in the post-operative period [8, 9]. Therefore, we chose to give a combined spinal epidural anesthesia to this patient. Regional anesthesia has the advantage of using a very limited number of drugs. Allergy to amide local anesthetics is rare [10]. Skin prick and intradermal tests with dilutions of the above-mentioned drugs was done pre-operatively and after confirming negative results, they were used [2]. Moreover, as the patient was conscious during regional anesthesia, anaphylaxis could be recognized earlier. The patient was followed for two months in the Nephrology Department and his post-operative stay was uneventful.
